# Decoding Partner Specificity of Opioid Receptor Family

**DOI:** 10.3389/fmolb.2021.715215

**Published:** 2021-09-21

**Authors:** Carlos A. V. Barreto, Salete J. Baptista, António J. Preto, Daniel Silvério, Rita Melo, Irina S. Moreira

**Affiliations:** ^1^ CNC - Center for Neuroscience and Cell Biology, University of Coimbra, Cantanhede, Portugal; ^2^ PhD Programme in Experimental Biology and Biomedicine, Institute for Interdisciplinary Research (IIIUC), University of Coimbra, Coimbra, Portugal; ^3^ Centro de Ciências e Tecnologias Nucleares, Instituto Superior Técnico, University of Coimbra, Coimbra, Portugal; ^4^ Department of Life Sciences, University of Coimbra, Coimbra, Portugal; ^5^ Center for Innovative Biomedicine and Biotechnology, Center for Neuroscience and Cell Biology, University of Coimbra, Coimbra, Portugal

**Keywords:** database, functional signature, GPCRs, opioid receptor, G-protein, arrestin

## Abstract

This paper describes an exciting big data analysis compiled in a freely available database, which can be applied to characterize the coupling of different G-Protein coupled receptors (GPCRs) families with their intracellular partners. Opioid receptor (OR) family was used as case study in order to gain further insights into the physiological properties of these important drug targets, known to be associated with the opioid crisis, a huge socio-economic issue directly related to drug abuse. An extensive characterization of all members of the ORs family (*μ* (MOR), *δ* (DOR), *κ* (KOR), nociceptin (NOP)) and their corresponding binding partners (ARRs: Arr2, Arr3; G-protein: G_i1_, G_i2_, G_i3_, G_o_, G_ob_, G_z_, G_q_, G_11_, G_14_, G_15_, G_12_, G_ssh_, G_slo_) was carried out. A multi-step approach including models’ construction (multiple sequence alignment, homology modeling), complex assembling (protein complex refinement with HADDOCK and complex equilibration), and protein-protein interface characterization (including both structural and dynamics analysis) were performed. Our database can be easily applied to several GPCR sub-families, to determine the key structural and dynamical determinants involved in GPCR coupling selectivity.

## 1 Introduction

G-Protein coupled receptors (GPCRs), the biggest family of membrane receptors, share a common three-dimensional structure: seven transmembrane domains (TM1-7) connected by three intracellular loops (ICL1-3) and three extracellular loops (ECL1-3). GPCRs also have an *α*-helix that runs parallel to the membrane in the intracellular side, commonly named Helix-8 (H8).

GPCR function is determined by the coupling with two main binding partners: heterotrimeric guanine nucleotide-binding proteins (G-proteins) and arrestins (Arrs). These receptors can interact with all four G_*α*_ classes (G_*αi*/*o*_, G_*αs*_, G_*αq*/11_ and G_*α*12/13_) that upregulate/downregulate distinct effectors. For example, G_*αi*/*o*_ inhibits cAMP production while G_*αs*_ increases its production, or G_*αq*/11_, which in turn activates protein phospholipase C and the phosphatidylinositol signaling pathway. Furthermore, Arrs can also activate G-protein independent signaling pathways or promote GPCRs internalization (Ma and Pei, 2007; Smith and Rajagopal, 2016). The experimentally determined three-dimensional (3D) structures helped decipher some GPCR-partner binding determinants. However, a detailed knowledge of the key interactions between GPCR and their partners has not yet attained. (Barreto et al., 2021 in press).

Arrestins consist of two retinal isoforms (visual arrestin (arrestin 1) and cone arrestin (arrestin 4), and two nonvisual arrestins, *β*-arrestin 1 (arrestin 2) and *β*-arrestin 2 (arrestin 3). The visual arrestins (arrestin 1 and 4) are confined to visual sensory tissue, *β*-arrestins are widely expressed. Therefore, arrestin 2 and 3 are the OR binding partners described in our study. For complexes with non-visual Arr, two different conformations were recently reported: one complexed with muscarinic two receptor (M2R) (PDB ID: 6U1N (Staus et al., 2020)), highly similar to the arrestin-rhodopsin complex (PDB ID:4ZWJ (Kang et al., 2015)), and another complexed with neurotensin receptor 1 (NTS1), in which Arr2 was shown to undergo a 90° rotation relative to the receptor. Therefore, and despite the lack of solved GPCR-Arr complexes, the data available so far suggests divergent bioactive Arr conformations. (Yin et al., 2019; Staus et al., 2020). Moreover, we now have new data that points to the possibility of major macromolecular complexes, involving the simultaneous binding to both Arrs and G-proteins (Nguyen and Lefkowitz, 2021). Although the number of available structures has quickly increased in the last decade, structures are often not available for an entire family of receptors and typically only cover one or two families of partners. Hence, computational approaches such as homology modeling, molecular dynamics and docking are still indispensable to generate reliable structural information of non-available receptors in both unbound and bound forms (Wang and Miao, 2019; Jaiteh et al., 2020).

Researchers in life science related disciplines (especially drug development) need to access, aggregate and analyze copious amounts of interdisciplinary data. The possibility to deliver digital infrastructures/platforms that can deliver such requirements is now a reality in the light of recent advances in big data analysis. Our in-house pipeline published in Preto et al. (2020), applied to the study of dopamine receptors, was further extended and its usefulness as a web-tool to generate a database that allows protein-protein interactions (PPIs) evaluation of key GPCRs sub-families was once again demonstrated by application to the characterization of the four opioid receptors (OR): *δ* (DOR), *κ* (KOR), *μ* (MOR), and nociceptin (NOP). This pipeline was created to circumvent the scarcity of experimental available GPCR-partner complexes structures and to generate extensive structural and dynamical data of these documented complexes (Preto et al., 2020). All generated data is available at http://www.moreiralab.com/resources/oxr.

## 2 Methods

### 2.1 Workflow

**FIGURE 1:**
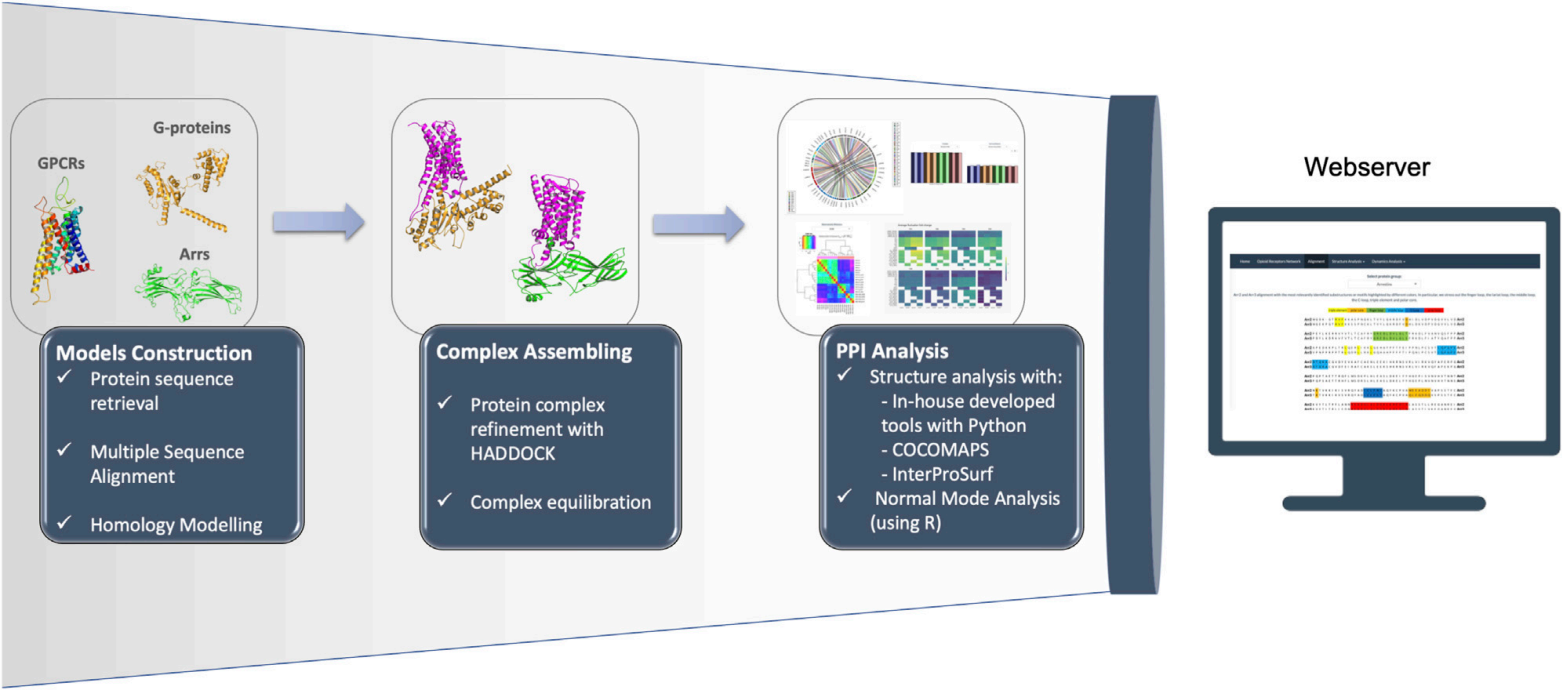
Representation of the OR pipeline.

Our GPCR PPI database is re-configurable in order to facilitate dynamical orchestration of operational components and provide a faster development/innovation lifecycle. The overall user workflow is available through a self-contained front-end application, which displays input information from compatible sources, and allows exploration of results. The resulting knowledge and full datasets are made freely accessible to its users. Also, the code to reproduce the analysis of the complexes is freely available at https://github.com/MoreiraLAB/or. We expanded our platform (Preto et al., 2020), to the OR family, for which all documented partners are described in Supplementary Table S1. The main pipeline steps, represented in Figure 1, are described below:

• Protein sequence retrieval from adequate database (e.g., Uniprot (The UniProt Consortium et al., 2021));
• Multiple Sequence Alignment (e.g., using Basic Local Alignment Search Tool (Altschul et al., 1990)) and biological inspection to pick adequate structural template;
• Homology modeling with Modeller (Šali and Blundell, 1993; Webb and Sali, 2016) or SWISS-Model (Waterhouse et al., 2018);
• Protein complex refinement with High Ambiguity Driven protein-protein DOCKing (HADDOCK) (van Zundert et al., 2016);
• Interface information retrieved using in-house Python (Van Rossum and Drake, 1995) tools and selenium (Software Freedom Conservancy, 2020), based on COCOMAPS (Vangone et al., 2011) and InterProSurf (Negi et al., 2007) servers;
• Additional features (amino acid and amino acid group percentages; 8 Å *α*-carbon distances; Salt-bridges; interhelical distance) calculated with in-house developed Python scripts (Van Rossum and Drake, 1995);
• Normal Mode Analysis performed using R (R Core Team, 2017);
• Hosting and webserver displayed with R (R Core Team, 2017) and shiny (Chang et al., 2021). Plots developed and displayed with ggplot2 (Wickham, 2016) and plotly (Plotly Technologies Inc., 2015).

#### 2.1.1 Constructing Models of Receptors and Partners

MODELLER package (Šali and Blundell, 1993; Webb and Sali, 2016) was used to generate 3D models of OR. Three distinct structures were used as templates to model the studied the receptors:

• the MOR-G_i_ complex (PDB-ID: 6DDF (Koehl et al., 2018));
• the M2R-Arr2 complex (PDB-ID: 6U1N (Staus et al., 2020));
• the NTS1-Arr2 complex (PDB-ID: 6PWC (Yin et al., 2019)).

MOR structure was used to model G-protein coupled conformation, while M2R and NTS1 were used to model two different Arr coupled conformations. Target sequences were extracted from UniProt (The UniProt Consortium et al., 2021) sequence IDs P41143, P41145, P35372 and P41146 for DOR, KOR, MOR and NOP, respectively. The sequence alignment, model building and model selection were performed according to Preto et al. (2020). Receptors’ models were embedded in a POPC:CHL1 membrane and subjected to an equilibration protocol, as described in Preto et al. (2020). The G_i_ structure from Rhodopsin-G_i_ complex (PDB-ID: 6CMO (Kang et al., 2018)) was used as template for partner models of G_i/o_, G_q/11_ and G_12_ subfamilies members. The G_slo_ and G_ssh_ model were built using as template the G_s_ structure retrieved from the *β*_2_AR-G_s_ complex (PDB-ID: 3SN6 (Rasmussen et al., 2011)). For Arr2 and Arr3 models, two distinct structure of Arr2 were used as template: one from NTS1-Arr2 complex (PDB-ID: 6PWC (Yin et al., 2019)) and the other from M2R-Arr2 complex (PDB-ID: 6U1N (Staus et al., 2020)). Human sequences of G_i1_, G_i2_, G_i3_, G_o_, G_ob_, G_z_, G_slo_, G_ssh_, G_q_, G_11_, G_14_, G_15_, G_12_, Arr2 and Arr3 were retrieved from UniProt (The UniProt Consortium et al., 2021) IDs P63096, P04899, P08754, P09471-1, P09471-2, P19086, P63092-1, P63092-2, P50148, P29992, O95837, P30679, Q03113, P49407-1 and P32121-1, respectively.

#### 2.1.2 Assembling Complexes

Models of receptors and partners were superimposed to templates using PyMOL (Schrödinger, 2015) and then refined with HADDOCK (van Zundert et al., 2016). HADDOCK software enables optimization of backbone and side chains at the complex interface, and as such the determination of the best possible model for each OR-partner complex. The complexes with G_i/o_, G_q/11_ and G_12/13_ members were superimposed on MOR-G_i_ structure (PDB-ID: 6DDF (Koehl et al., 2018)). The OR-G_q/11_ and OR-G_12/13_ complexes were also superimposed on M1R-G_11_ structure (PDB-ID: 6OIJ (Maeda et al., 2019)). The complexes with G_s_ members were superimposed with *β*_2_AR-G_s_ complex (PDB-ID: 3SN6 (Rasmussen et al., 2011)). Arrestin complexes were superimposed on NTS1-Arr2 complex (PDB-ID: 6PWC (Yin et al., 2019)) and M2R-Arr2 complex (PDB-ID: 6U1N (Staus et al., 2020)).

#### 2.1.3 Analysis of the Complexes

As our previous work show, detailed PPI characterization is fundamental to shed light into complex biological mechanisms (Preto et al., 2020). Some of the key elements to understand these problems (e.g., GPCR-partner coupling), implies measurement of structural features like interacting residues, number of h-bonds (HB) and salt bridges (SB) and solvent accessible surface area (SASA) analysis, as well as an atomistic characterization of their dynamic behavior. As such, normal mode analysis (NMA), to investigate flexibility and fluctuation changes between receptor structure in monomer and in complex, as well as distance determination between TM domains, to characterize GPCR activation, were also implemented.

### 2.2 Server Architecture

The OR portal can be accessed by modern popular Web browsers, including Chrome, Internet Explorer, Safari, and Firefox, without installing any specialized software or browser plug-ins. The implementation was performed using shiny (Chang et al., 2021) within R (R Core Team, 2017). In the view tier, the front-end plots were developed using ggplot2 (Wickham, 2016), for static information, and embedded with plotly (Plotly Technologies Inc., 2015), when possible, for a more intuitive and dynamic analysis. The back-end deployment was performed and hosted in our own servers.

## 3 Case Study: Interface Characterization of OR-Partners

**FIGURE 2:**
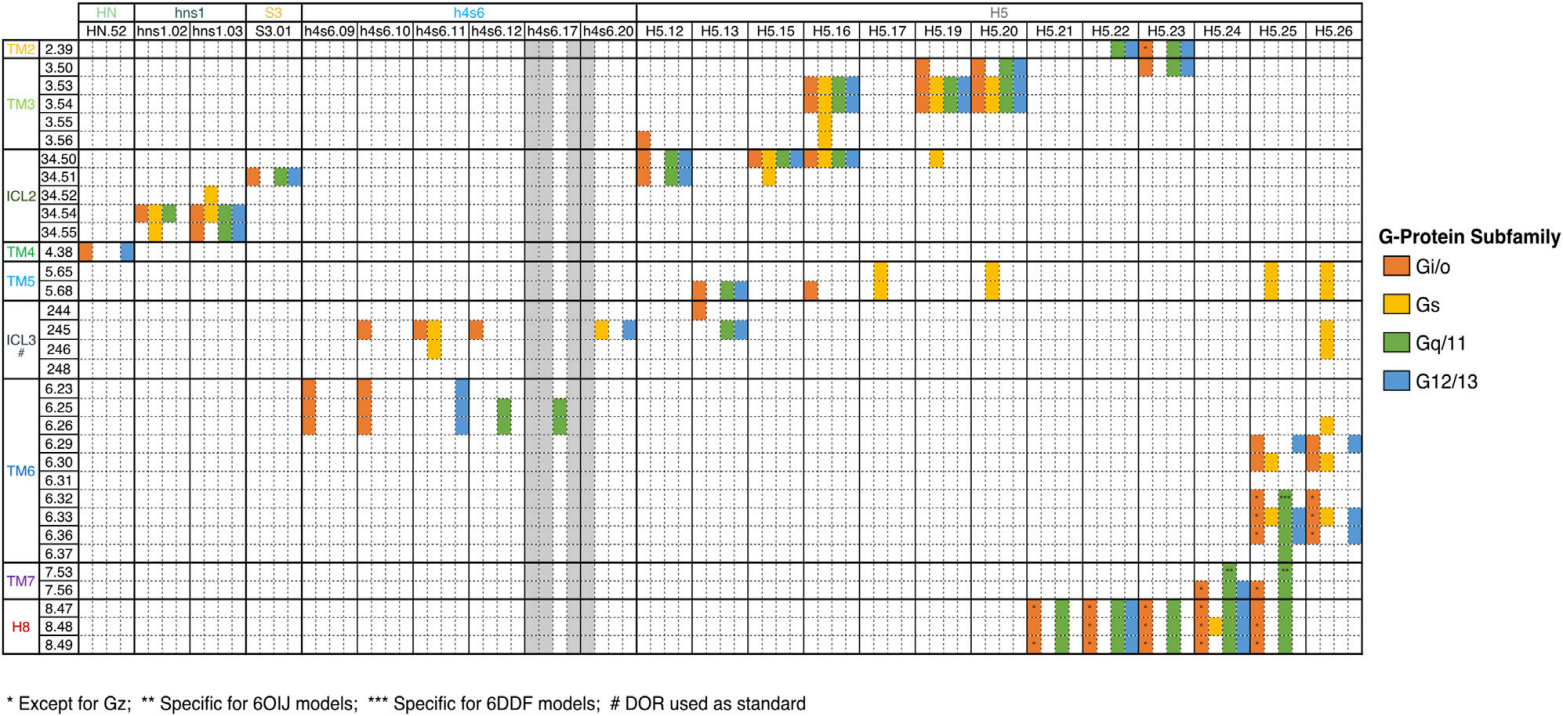
Key interactions between OR and G-Proteins. Only common interactions between the four receptors were depicted. The Ballesteros-Weinstein (BW) numbering scheme, which allows the comparison of any amino acid residue in the structurally conserved TM helices of GPCRs, was used. For ICL2, residues were labeled 34. X, indicating the positions between TM3 and TM4. X.50 was once again attributed to the most conserved residue. DOR residues numbers were used for ICL3 numbering, since this substructure is not conserved among OR.

**FIGURE 3:**
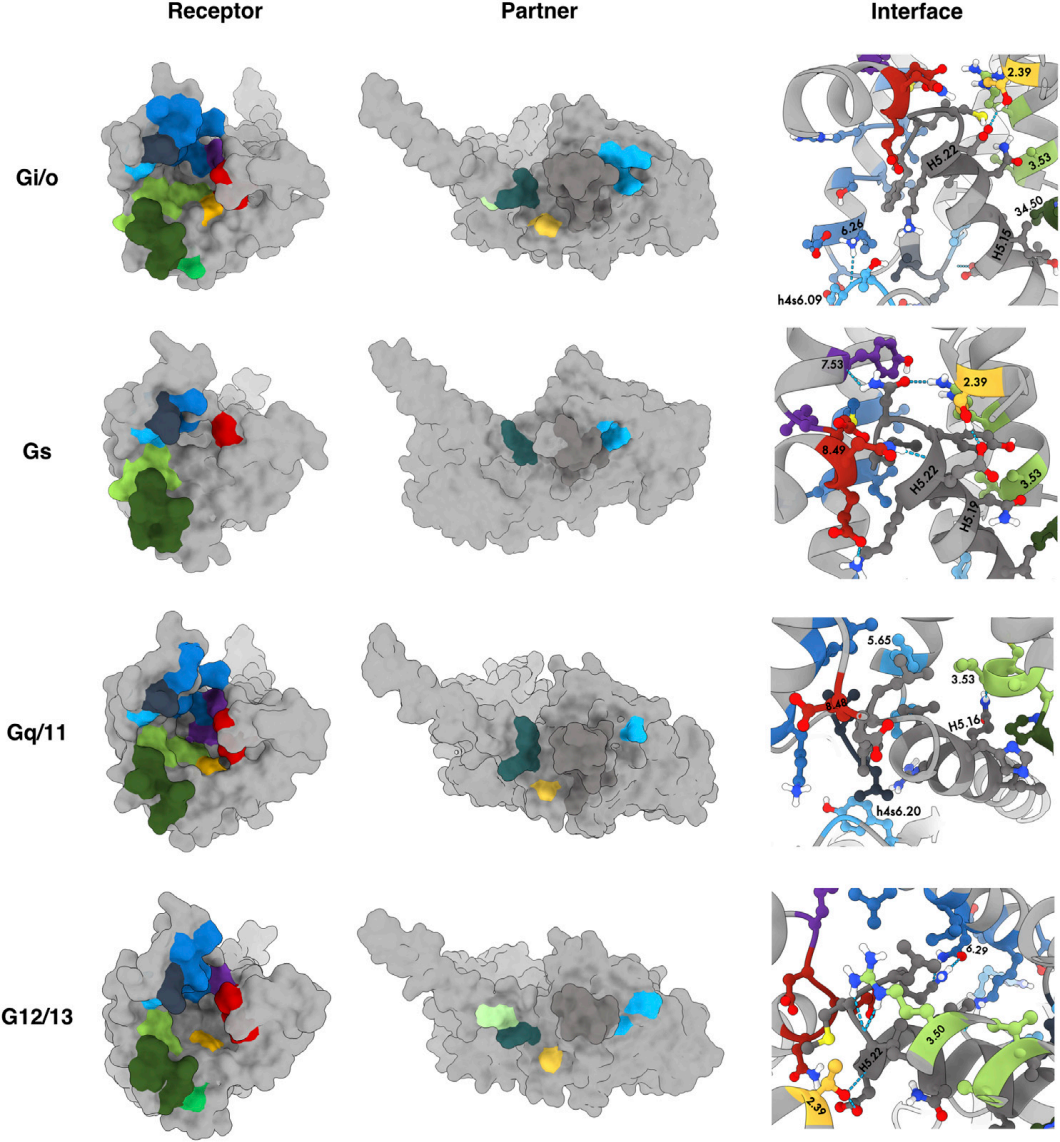
Common interaction binding motifs in the OR family. Receptors and partners are both represented as surface. 3D structures correspond to DOR-G_i1_, KOR-G_ssh_, DOR-G_q_ and KOR-G_12_ (from **top to bottom**). Residues reported in Figure 2 were colored by their subdomain using the color scheme available at the sequence alignment on the webserver. Receptor: TM2 - yellow; TM3 - light green; ICL2 - dark green, TM4 - lime; TM5 - light blue; ICL3 - dark blue; TM6 - blue, TM7 - purple; H8 - red. Partner: HN - light green, hns1 - dark teal; S3 - yellow; h4s6 - light blue; H5 - dark grey.

In our previous work, we have already detected significant signatures for the differential coupling of dopamine receptors, alongside a detailed characterization of all involved partners (Preto et al., 2020). Herein, we constructed a dedicated database focused on the OR family. Our pipeline was able to capture and reproduce the known data as well as infer new possibilities, that could be tested by the scientific community. In the next subsection, we detail the main biological information retrieved for the OR complexes. A summary of the interacting residues is provided in a heatmap-like figure **(** Figure 2 **)** and by a 3D structural representation of receptor’s and partner’s interface **(** Figure 3 **)**. A more detailed discussion of the test case can be found at SI.

Binding motifs were named using one-code amino acid nomenclature and in agreement with Ballesteros-Weinstein numbering scheme (Ballesteros and Weinstein, 1995), which defines X.50 as the most conserved residue in each TM domain or the residue number in case of belonging to an ICL or the binding partner in superscript. A wild-card amino acid was defined using “x”. The analysis of the main interactions established between OR and G-proteins pointed out some interesting interaction patterns, involving ICL2, TM3, TM5, ICL3 and TM6, which are in line with the MOR-G_i_ complex structure determined by Koehl et al. (2018). Particularly, **P** ^**34.50**^ **–V/I** from **P** ^**34.50**^ **–V/I–x**_**2**_ **LD** ^**34.55**^ pattern (ICL2) interacts with a hydrophobic patch of G_i/o_ and G_12_, which is constituted by **T/K** ^**H5.12**^ **–x**_**2**_ **I–I/L** ^**H5.16**^ pattern from H5 and **L/I** ^**S3.01**^ from S3 **(** Figure 2 **)**. Although this hydrophobic pattern is not conserved for G_q/11_ and G_s_, ORs are still interacting through **P** ^**34.50**^ **V** with the corresponding residues at H5 and S3 positions. Moreover, the **D** ^**34.55**^ (ICL2) interacts with **R/K/G/A** ^**hns1.03**^ of hns1, in all OR-G-proteins complexes (except for KOR-G_s_). Even though **D** ^**34.50**^ is a conserved residue among OR family, the same does not apply to other G_i_-protein primary coupling GPCRs, such as D_2_R where the above-mentioned interaction is not observed (Koehl et al., 2018; Preto et al., 2020; Yin et al., 2020). Some hydrophobic residues from TM5 (**V** ^**5.68**^) and ICL3 (**L** ^**245/258/247**^ **/M** ^**266**^) were also involved in interactions with G-proteins’ H5, in a similar way to what was previously reported by Koehl et al. (2018). It is also worth mentioning that **R** ^**244/257/265/246**^, an important residue in G_i_ signaling, was found to be engaged in interactions not only with G_i_, but also with G_q/11_ and G_12_ subfamilies, in most of OR-complexes, through H5 (**D** ^**H5.13**^) and/or h4s6 (e.g., **E** ^**h4s6.12**^ (G_i/o_), **I/V/R/P** ^**h4s6.20**^ (G_q/11_ and G_12_ subfamilies)). This interaction is not observed for OR-G_s_ complexes, which agrees with previous data showing that an identical interaction was not present in the *β*_2_AR-G_s_ complex (Rasmussen et al., 2011; Koehl et al., 2018). Furthermore, the cytosolic ends of TM3 and TM6 were engaged in interactions with highly conserved residues at H5 C-terminal. Similarly to the information reported for MOR-G_i_ complex by *Koehl et al.*, TM3 (mostly through the conserved **R** ^**3.50**^), interacted with the G_i/o_ conserved **C** ^**H5.23**^. This interaction mediated by TM3 was not observed for OR when complexed with G_z_ (**I** ^**H5.23**^), or KOR-G_s_ complexes. Nevertheless, a similar interaction between **R** ^**3.50**^ and **Y/F/I** ^**H5.23**^ was also observed for OR-G_q/11_/G_12_ or MOR-G_s_ complexes. The fully conserved **L** ^**H5.25**^, from C-terminal H5, also interacts with a hydrophobic pocket formed by TM6 residues such as **I** ^**6.33**^, **M/L** ^**6.36**^, and **V** ^**6.37**^, for OR upon coupling to G_i/o_ and G_q/11_ subfamilies, as well as for MOR-G_s_, data corroborated by literature (Koehl et al., 2018). An additional interaction between **L** ^**H5.25**^ and **R** ^**6.32**^, can further stabilize the interface of interaction in OR-G_i/o_ and OR-G_q__6DDF complexes, particularly for DOR, MOR and NOP ones. Through the interaction plot analysis, we also disclosed that **T** ^**2.39**^ was relevant in all OR-G-protein complexes, with the exception of MOR-G_o_, G_s_ or G_z_, where this residue (or any other TM2 residue) did not mediated any interaction. The interaction between **T** ^**2.39**^ and the **C/Y/F/I** ^**H5.23**^ (H5) was a characteristic one for G_i/o_ and G_q_ complexes. This fact agrees with our previous findings, in which both G_i/o_ and G_q_ complexed with DXR revealed this same interaction (Preto et al., 2020). On the contrary, no interaction involving TM2 was shown in OR-G_s_ or DXR-G_s_. Another interesting sequence pattern highlighted in the present interface characterization study involves both TM7 and H8, through **L** ^**7.56**^ **–D** ^**8.47**^ **EN** ^**8.49**^ interacting motif, and G_i/o_, G_12_ and G_q/11_ subfamilies. This interaction pattern was not, however, observed for either G_s_ or G_z_ complexes that did not interacted with OR TM7 subdomain. These results also follow previous reported data demonstrating a higher number of interactions involving the TM7-H8 boundary in G_i_-coupled receptors, when compared with G_s_-coupled ones (Draper-Joyce et al., 2018; Sandhu et al., 2019). This is also in line with our previous study, in which we identified the interacting pattern **F** ^**7.56**^ **N–A/I–E** ^**8.45**^ for G_i/o_ and G_q_ couplings with D_2_-like Receptors (Preto et al., 2020). The recent solved D_2_R-G_i_ structure also suggested the interaction of TM7-H8 junction (namely **F** ^**7.56**^, **N** ^**8.43**^ and **E** ^**8.45**^) with the C-terminal H5 interface of G_i_ (Yin et al., 2020).

Regarding OR-Arrs interactions analysis, some interesting features were also revealed. One of the most remarkable differences between OR-Arrs_6U1N and OR-Arrs_6PWC was the number of interactions established between OR and their intracellular effectors. Clearly, the latter complex group had a smaller number of interactions comparing with the former one. This is in accordance with dopamine complexes data (Preto et al., 2020), and could be linked to the 90° rotation of Arr2 in NTS1-Arr2 (PDB-ID: 6PWC (Yin et al., 2019)) structure when compared with M2R-Arr2 (PDB-ID: 6U1N (Staus et al., 2020)). The finger loop was the most interacting subdomain for both OR-Arrestins_6U1N and OR-Arrestins_6PWC groups. We observed that both Arrs interacted through a more embracing finger loop motif (**Y** ^**63/64**^ **–G** ^**64/65**^ **x**_**4**_ **DVLGL** ^**73/74**^) in OR-Arrs_6U1N complexes when compared with the OR-Arrestins_6PWC ones (**D** ^**67/6**8^ **x**_**2**_ **VL** ^**71/72**^). This follows the differences found in both templates (NTS1-Arr2 and M2R-Arr2). In the M2R-Arr2, the finger loop had a higher number of interacting residues and contacts extensively with TM2, TM3, ICL2, TM5, and TM6. Like the template, two interactions appears to be conserved in all OR-Arrestins_6U1N and include the DRY motif (TM3) residues: the interaction between **D** ^**69/70**^ and **T** ^**2.39**^ or **V** ^**70/71**^ and **V** ^**3.50**^. The change of **R** ^**3.50**^ (in NTS1) by a **V** ^**3.50**^ (in OR) does not significantly affect the interaction profile (Staus et al., 2020). Moreover, M2R-Arr2 also reports a special positioning of ICL2 inside a hydrophobic cleft between the two Arrs domains. The results obtained for OR-Arrs_6U1N complexes, showed that ICL2 interacts with residues from both domains endorsing the authors conclusions (Staus et al., 2020). Concerning OR-Arrs_6PWC, it was observed that TM6, TM7 and H8 were always involved in interactions with finger loop, in line with the solved NTS1-Arr2 structure (Yin et al., 2019). Furthermore, TM3 and TM5 also contacted the finger loop at KOR-Arrs_6PWC complexes. The same was not observed for the NTS1-Arr2 complex (Yin et al., 2019).

Another interesting difference between OR-Arrs_6PWC and the original modeling template can be pointed out. While ICL1 was an interacting domain in NTS1-Arr2, particularly with lariat and bottom loops, in OR-Arrestins_6PWC, both Arrs only interacted with ICL1 in three complexes (MOR-Arr2_6PWC and NOP-Arrs_6PWC), all of them through the finger loop. On the other hand, the ICL2 formed contacts in all complex models, establishing interactions with finger loop and C-loop, instead of the lariat loop as reported in the template. This draws the hypothesis that ICL2 is not deeply inserted into the reported cleft (with middle loop, bottom loop and lariat loop), present in Arrs, interacting with finger loop at the intracellular cavity. It is also noteworthy that the ICL3 interaction pattern was in line with the NTS1-Arr2 structure, although only one interacting ICL3 residue was involved in establishing protein-protein contacts, for all OR-Arrs_6PWC complexes. This was a significant difference regarding arrestin recognition, when compared to other GPCRs with long ICL3, where multiple ICL3 residues interact with the receptor (Kang et al., 2015; Yin et al., 2019). On the other hand, some interactions previously described by *Goddard et al.* Mafi et al. (2020). were not observed in this study, namely the interaction between **R** ^**34.57**^ (ICL2) and **R** ^**65/66**^ (finger loop). **R** ^**34.57**^ (ICL2) was reported as a key residue in Arr-2 binding regulation, mediating the creation of a polar network interactions which stabilizes the active-state of Arr2-pp-KOR complex bound to the full agonist DAMGO. Its interaction with **R** ^**65/66**^ allowed the coordination of the later residue, which is thus able to establish a salt-bridge with the **D** ^**67/68**^. This promote the reorientation of **D** ^**67/68**^ in order to form a hydrogen bond with the TM2. (Mafi et al., 2020).

Since our published work on DxR complexes, experimentally determined complex structures of D_1_R-G_s_ (PDB-ID: 7JVP (Zhuang et al., 2021b), 7JVQ (Zhuang et al., 2021b), 7JV5 (Zhuang et al., 2021b), 7LJD (Zhuang et al., 2021a), 7LJC (Zhuang et al., 2021a), 7CKY (Xiao et al., 2021), 7CRH (Xiao et al., 2021), 7CKW (Xiao et al., 2021), 7CKX (Xiao et al., 2021), 7CKZ (Xiao et al., 2021)) and D_2_R-G_i_ (PDB-ID: 6VMS (Yin et al., 2020), 7JVR (Zhuang et al., 2021b)) have been released. The similarly between our complexes and the experimental reported is very high, with RMSD values ranging from 1 Å to 1.5 Å. Xiao et al. (2021) reported 16 different contacts between D_1_R-G_s_ structure and D_2_R-G_i_ structure. D_1_-like receptors have a Phe **(F** ^**34.51**^) while D_2_-like have Met **(M** ^**34.51**^) and Leu **(L** ^**34.52**^) at the ICL2, which interacts with an hydrophobic pocket created by S1, S3 and H5 of G_s_ (Xiao et al., 2021). This interaction extends to other residues from ICL2 **(P** ^**34.50**^ **, R** ^**34.52**^ **, E** ^**34.54**^ and **R** ^**34.55**^). In Preto *et al.*, where we used a similar protocol, we were able to correctly predict the same Phe interaction with G_s_ in D_1_-like receptors inside **P** ^**34.50**^ **Fx**_**2**_ **–E/K–R** ^**34.55**^ pattern (Preto et al., 2020). Another important interaction between D_1_R-G_s_ was predicted in Preto *et al.* and identified in Xiao *et al.* at TM5 (**A** ^**5.65**^ **xxQ** ^**5.68**^ **-I** ^**5.69**^).

## 4 Conclusion

Our integrated end-to-end engine platform provides access to the putative protein-protein interfaces and key pairwise interactions involved in the coupling of OR receptors and G-proteins and/or Arrs partners. Although the modeling and web server can be assembled in a system independent way, we applied it to a particular relevant sub-family as we aim to link our platform to a careful analysis of all data in a consistent and integrative way.

Our pipeline was successful in predicting the interactions reported for previous solved GPCR-Partners complexes, disclosing the main differences engaged in the coupling of the distinct G-proteins subfamilies, as well as the different Arrs conformations involved. Our demonstrated success upon application to two different GPCR sub-families (DxR and OR), demonstrates the protocol scalability giving non-expert researcher a tool to quickly analyze and assess the interface between a receptor and its intracellular partners.

## Data Availability

The datasets generated and analyzed for this study can be found in http://www.moreiralab.com/resources/oxr. The platform is free and open to all. There is not no login requirement. The code and models used to generate the datasets are fully available at https://github.com/MoreiraLAB/or.
