# Supplementary material for: Decoding Partner Specificity of Opioid Receptor Family
Source: Front Mol Biosci. 2021 Sep 21;8:715215. doi: 10.3389/fmolb.2021.715215 (PMC8490921; doi:10.3389/fmolb.2021.715215)
Supplement: Supplementary file 1 [file DataSheet1.pdf]

Decoding partner specificity of opioid receptor family

1 SUPPLEMENTARY DATA

1.1 Structural Features

1.1.1 Interhelical Distances

In order to understand the opening of the intracellular cavity in the different complexes, the distances between TM3-TM6 and TM3-TM7 were plotted against each other as applied in *Sandhu et al.* (Sandhu et al., 2019) and *Preto et al.* (Preto et al., 2020). The TM3-TM6 distance for G-protein complexes ranges between 14 and 16 Å whereas the TM3-TM7 ranges between 12 and 14 Å. Complexes were grouped by receptor across the TM3-TM7 axis, and DOR and NOP showed a lower crevice opening (12-13 Å) followed by MOR and finally KOR that presented the biggest distances between TM3 and TM7. On the TM3-TM6 axis, the systems were all clustered around 14-15 Å, except for MOR-G_s complexes that showed the highest TM3-TM6 distance. No differences between G-protein subfamilies were observed along TM3-TM7. On the TM3-TM6 axis, the MOR-G_s has shown a different behavior from the remaining G_i complexes, presenting the former a higher TM3-TM6 distance when compared with the later ones. This difference was not observed for G_s-bounded KOR complexes, which were similar to the remaining KOR complexes. (**Figure S1-A**) Arrestin complexes presented higher variations of the two measured distances when compared to G-Protein complexes, suggesting a higher crevice opening for OR-Arrs complexes. TM3-TM6 distance ranged between 15-18 Å and TM3-TM7 distances ranged between 11-16 Å. Complexes modeled with 6PWC showed consistently lower TM3-TM6 distances as well as TM3-TM7 distances, except for NOP complexes that presented identical TM3-TM7 distances between 6PWC and 6U1N derived models (**Figure S1-B**).

1.1.2 Interface Percentage

The percentage of each amino acid at the receptors' and partners' surfaces was also calculated. Looking at the OR-Arrs complexes, nonpolar aliphatic residues were the most predominant (particularly Leu) at the partner interface, followed by basic positive (Arg) for 6PWC complexes and acid negative (Asp) for 6U1N complexes. At the receptor's surface, the most predominant amino acid group was shown to be the nonpolar aliphatic for both templates. The most predominant amino acid was clearly different between templates: Arg was enriched in 6PWC and Leu in 6U1N, except for NOP complexes where Arg was the most predominant one. Gln and Trp were both absent in receptor's and partner's interfaces. Gly, Cys and His (except for NOP-Arr3_6PWC) were only present at the receptor's interface in the 6U1N models. The same happened for Ile and Cys at partner's interface. In the partner's side of the interface, Asn appeared solely on Arr2 complexes, while Ser only appeared at Arr3 complexes.(**Figure S2**). Looking at $G_{i/o}$ family, nonpolar aliphatic was still the most predominant group at both partner's and receptor's interfaces, followed by acid negative residues at G-protein interface and basic positive at receptor's interface. The only exceptions to this observation were the G_o and G_{ob} complexes that showed more basic positive residues in the partner's interface than acid negative. Asp was the most predominant residue in G_{i1}'s and G_{i2}'s interfaces, while in G_{i3}, Asp, Glu, Leu and Lys had identical percentages. G_o and G_{ob} showed a interface

rich in Lys. G_Z was the only member of the family enriched in Glu instead of Asp. On the receptor's side, the environment was highly positive due to the higher percentages of Arg. Phe, Trp, Cys and Gln were absent from the receptor's interface, whereas Met and Trp were absent from G-protein's interface. Pro was only present in G_o complexes, while G_Z was the only complex without any Cys. In OR-G_s complexes, both interfaces were enriched in basic positive residues, as well as in polar uncharged groups, particularly true for MOR-Complexes.

1.2 Patterns of Interaction

Almost all OR subdomains were involved in establishing key contact points with Arrs and/or G-proteins. The only exceptions were observed for TM4, which did not engage with Arrs, and for TM1, which did not interact with any Arrs or G-proteins. Concerning OR-Arrs complexes, TM2, TM3, ICL2, TM5 and TM6 were the prevalent interacting OR subdomains in complexes modeled with 6U1N, while ICL2, TM6 and H8 subdomains interacted with Arrs modeled with 6PWC. TM2 interacted mainly with the Arrs finger (through residue $\mathbf{T^{2.39}}$) in almost all complexes, except for DOR-Arrs_6PWC and KOR-Arr3_6PWC complexes. Furthermore, TM3 appears to be involved in PPIs, particularly at OR-Arrs_6U1N complexes, through the interaction pattern $\mathbf{R^{3.50}x_2A\text{–}V/I^{3.54}}$. The finger loop was indeed key for all PPIs at Arrs_6U1N subdomain, except for NOP-Arr3_6U1N, in which it was substituted by interaction of the C-loop with TM3. For OR-Arrs_6PWC complexes, TM3 only interacted with KOR-Arrs_6PWC complexes through $\mathbf{AV^{3.54}}$ residues.

All OR-Arrs complexes showed similar interaction patterns at ICL2, namely $\mathbf{V/I^{34.51}x_2LD^{34.55}}$ (OR-Arrs_6PWC) and $\mathbf{P^{34.50}\text{–}V/I\text{–}K/R\text{–}x_2D^{34.55}}$ (OR-Arrs_6U1N). Nevertheless, while C-loop ($\mathbf{F^{244/245}\text{–}N/S}$ motif) was a common interaction for all OR-Arrs_6PWC, for OR-Arrs_6U1N complexes, the ICL2 interacted with the finger and lariat loops ($\mathbf{R^{285/286}G}$ motif). It is noteworthy that the finger loop was, however, engaged in interactions with ICL2 for KOR and MOR-Arr2_6PWC complexes. Moreover, TM5 ($\mathbf{R^{5.63}}$ or $\mathbf{R^{5.64}}$) interacted with the C-terminal ($\mathbf{F^{244/245}}$) and finger loop in all OR-Arrs_6U1N complexes. However, this subdomain was not relevant for OR-Arrs_6PWC complexes, with the exception of KOR-Arrs_6PWC. ICL3 was also engaged in pairwise interactions in all OR-Arrs complexes, except in NOP-Arrs_6PWC and KOR-Arrs_6U1N complexes. TM6 was found to mainly interact with the finger loop, through a common residue ($\mathbf{S/N^{6.29}}$). Despite interacting only through $\mathbf{L^{7.56}}$, TM7 also mediated interactions in OR-Arrs complexes, with the exception of MOR-Arrs_6U1N and NOP-Arr3_6PWC. $\mathbf{D^{8.47}}$ was relevant for OR-Arrs_6U1N (except KOR-Arrs_6U1N and MOR-Arr3_6U1N), while $\mathbf{D^{8.47}E}$ residues interacted in OR-Arrs_6PWC complexes. From the Arr side, beyond the already mentioned interactions with the lariat and C-loops, the majority of the interactions were established through the finger loop. $\mathbf{D^{67/68}x_2VL^{71/72}}$ and $\mathbf{G^{64/65}x_4DVLGL^{73/74}}$ were the common interaction patterns involved for OR-Arrs_6PWC and OR-Arrs_6U1N, respectively. In all cases, the finger loop interacts with several OR subdomains, simultaneously.

Regarding OR-G-protein complexes, most of the TMs were present at the established interfaces (except for TM1), alongside ICL2 and ICL3. TM3, ICL2, TM6 and H8 contacted with all G-proteins for all OR-complexes, with no exception. Moreover, specific interaction patterns were disclosed involving these interacting substructures, such as $\mathbf{P^{34.50}\text{–}V/I\text{–}x_2LD^{34.55}}$ for all OR-G-protein complexes (except for KOR-G_{slo} that interacted through $\mathbf{P^{34.50}VK^{34.52}}$). On the other hand, and while the common interaction pattern $\mathbf{R^{3.50}x_2AVxH^{3.56}}$ was found to DOR and MOR when complexed with $G_{i/o}$ subfamily, only $\mathbf{A^{3.53}\text{–}V/I}$ residues interacted with these partners at KOR and NOP receptors. For G_{12} and G_q complexes, the pattern $\mathbf{R^{3.50}x_2A\text{–}V/I^{3.54}}$ was maintained for all OR complexes but MOR-G_q_6DDF. $\mathbf{A^{3.54}\text{–}V/I\text{–}CH^{3.56}}$

was a common interacting motif for KOR-G_s complexes and it was extended to $\mathbf{R^{3.50}x_2A–V/I–CH^{3.56}}$ to MOR-G_s complexes. $\mathbf{T^{4.38}}$ was involved in one interaction with G-proteins' HN ($\mathbf{E/I/Q^{HN.52}}$) for all KOR complexes but KOR-G_s. This contact was observed in other OR-complexes, with exception of G_s, in which it did not occur at all. TM5 interacted through $\mathbf{V^{5.68}}$ with all G-proteins, except in the DOR-G_{15}_6OIJ case. For KOR-G_z and KOR-G_s complexes, $\mathbf{L^{5.65}x_2V^{5.68}}$ were involved in interfacial interactions. MOR-G_s complexes were the ones where a higher number of TM5 residues were present at the various PPIs. Moreover, ICL3 was engaged in interactions in all OR-complexes, except for MOR-G_{15}_6OIJ. The main interacting domain on the OR side was TM6, which particularly interacted with $G_{i/o}$ and G_{12} subfamilies through the common interaction pattern $\mathbf{S^{6.23}xEKx_2–S/N–x_3Ix_2–M/L^{6.36}}$ (except for G_z). This pattern was extended to $\mathbf{S^{6.23}xEK\ x_2–S/N–LxRIx_2–M/L–V^{6.37}}$ for DOR, MOR and NOP when complexed with $G_{i1\text{-}3}/G_o/G_{ob}$. $\mathbf{K^{6.26}x_6Ix_2–M/L^{6.36}}$ was also an interaction pattern for OR-$G_{q/11}$ subfamily (except for DOR-G_q_6OIJ). In OR G_q_6DDF complexes, a higher number of TM6 residues were engaged in contacts with the partner, with $\mathbf{K^{6.26}x_5RIx_2–M/L^{6.36}}$ as a common interaction motif for all G_q modeled and aligned with 6DDF. Furthermore, $\mathbf{L^{7.56}}$ was involved in interaction for all G-proteins but G_z or G_s. Finally, H8 was also a key motif in all OR-G-protein complexes, through the common interaction motif $\mathbf{D^{8.47}EN^{8.49}}$ for all OR complexed with $G_{i1\text{-}3}/G_o/G_{ob}$, G_{12} and NOP-$G_{q/11}$_6DDF subfamily. For DOR and MOR complexed with $G_{q/11}$ proteins aligned with 6DDF, alongside DOR-G_z and MOR-G_s, only $\mathbf{D^{8.47}E^{8.48}}$ interacted with H5. For G_z (namely KOR, MOR and NOP receptors) and KOR-G_s complexes, only $\mathbf{E^{8.48}}$ was able to contact with that substructure. For NOP-$G_{q/11}$_6OIJ complexes subfamily, the interacting motif was extended to $\mathbf{D^{8.47}ENF^{8.50}}$. On the G-proteins side, H5 was the structural motif with the most number of contacts with OR, interacting with TM2, TM3, ICL2, TM5, TM6, TM7 and H8. $\mathbf{T^{H5.12}DxIIx_2Nx–K/R–D/E/G–CGL–Y/F^{H5.26}}$, $\mathbf{K/R^{H5.12}–Dx–I/V–Lx_2–N/Y–x_3–Y/F/I–NL^{H5.25}}$, and $\mathbf{I^{H5.15}QRxHLx_3ELL^{H5.26}}$ were the common H5 interaction motifs for G_i (except G_z), G_q and G_s when complexed with OR. Another interacting pattern observed for the different OR-G-protein subfamilies involved h4s6 motif, namely $\mathbf{T/N/H^{h4s6.10}KE^{h4s6.12}}$ (for G_i), $\mathbf{D/E^{h4s6.12}–K–I/V/R^{h4s6.20}}$ (for Gq_6DDF), and $\mathbf{S^{h4s6.02}TASGDGx_2Y^{h4s6.20}–C^{S6.01}}$ (for MOR-G_s). In this case, the pattern includes $\mathbf{C^{S6.01}}$, a S6 residue, only involved in interactions with G_s complexed with MOR). h4s6 engaged in contacts with ICL3 and TM6 for the large majority of OR-complexes. However, some exceptions can still be pointed out. For DOR-G_{14}, DOR-G_{15} and MOR-G_{15}, when aligned with 6OIJ template, h4s6 only established interactions with TM6. Conversely, in KOR-G_s complexes, only contacts between h4s6 and ICL3 were observed. Interestingly, KOR residue $\mathbf{R^{257}}$ (ICL3) interacted with $\mathbf{E^{h4s6.12}}$ in all $G_{i/o}$ subfamily members. An interaction between $\mathbf{R^{257}}$ and $\mathbf{P^{h4s6.20}}$ was also observed for KOR-G_{12}_6DDF, which was not verified for G_{12} modeled with 6OIJ, or KOR-G_s complexes.

1.3 Dynamical Features

Normal Mode Analysis considering only C-α was performed in monomeric and complex structures of all ORs. Changes in flexibility and fold changes in fluctuation were computed for all relevant substructures as described in Preto *et al.* (Preto et al., 2020). OR-Arrestins and OR-G_s showed distinctively low average fluctuations values when compared with $G_{i/o}$, G_q and G_{12} complexes. The latter showed high average fluctuation values, in particular for TM1 through TM4 and TM7. (**Figure S3-A**)

The flexibility changes of relevant substructures were calculated as the Bhattacharya coefficients (BC) between monomeric and complex structures. This acts as a coefficient of similarity, where high similarity is indicated by values close to 1 (Preto et al., 2020). OR-Arrestins and OR-G_s displayed slightly higher BC values than the rest of the complexes, particularly at TM4, TM5 and TM6. $G_{i/o}$, $G_{q/11}$ and G_{12} complexes,

showed lower values of BC, especially at TM1, TM5 and TM6. KOR-$G_{i/o}$ and KOR-G_{12} are the exception, with high BC values at TM1. H8 structure has the highest BC values for all complexes (**Figure S3-B**). The multidimensional scaling map showed a very clear distinction between OR-Arrestins/OR-G_s and OR-$G_{i/o}$/OR-$G_{q/11}$. KOR-$G_{i/o}$ and KOR-G_{12} complexes are isolated from the rest of these partners' cluster. DOR-Arrs_6U1N were also completely distant from their respective group (**Figure S3-C**). The dynamic analysis results demonstrated some differences between different OR-Partner subgroups. In general, the coupling between OR and $G_{i/o}$/OR-$G_{q/11}$/G_{12} promotes higher dynamical changes in OR structures than in OR-G_s or OR-Arrestins. Similar results were found in the dopamine receptors (Preto et al., 2020).

2 SUPPLEMENTARY TABLES

Table S1. Functional Binding Partners of the OR Family

Receptor		DOR	KOR	MOR	NOP
G-Protein	Gi1	(Inoue et al., 2019)	(Inoue et al., 2019)	(Chan et al., 2002; Inoue et al., 2019; Burford et al., 1998; Sánchez-Blázquez et al., 2001)	(Inoue et al., 2019)
	Gi2	(Inoue et al., 2019)	(Inoue et al., 2019)	(Chan et al., 2002; Inoue et al., 2019; Burford et al., 1998; Sánchez-Blázquez et al., 2001)	(Inoue et al., 2019)
	Gi3	(Inoue et al., 2019)	(Inoue et al., 2019)	(Chan et al., 2002; Inoue et al., 2019; Burford et al., 1998; Sánchez-Blázquez et al., 2001)	(Inoue et al., 2019)
	Go	(Inoue et al., 2019)	(Inoue et al., 2019)	(Chan et al., 2002; Inoue et al., 2019; Burford et al., 1998; Sánchez-Blázquez et al., 2001)	(Inoue et al., 2019)
	Gob	(Inoue et al., 2019)	(Inoue et al., 2019)	(Chan et al., 2002; Inoue et al., 2019; Sánchez-Blázquez et al., 2001)	(Inoue et al., 2019)
	Gz	(Inoue et al., 2019)	(Inoue et al., 2019)	(Chan et al., 2002; Inoue et al., 2019; Sánchez-Blázquez et al., 2001)	(Inoue et al., 2019)
	Gs	—	(Hampson et al., 2000)	(Wang et al., 2016; Szücs et al., 2004)	—
	Gq	(Narita et al., 2000)	—	(Sánchez-Blázquez et al., 2001)	(Lou et al., 1997)
	G11	(Narita et al., 2000)	—	(Sánchez-Blázquez et al., 2001)	(Lou et al., 1997)
	G14	(Inoue et al., 2019)	—	—	(Inoue et al., 2019; Yung et al., 1999)
	G15	(Inoue et al., 2019; Zhu et al., 2008)	—	(Offermanns and Simon, 1995)	—
	G12	—	(Inoue et al., 2019)	—	(Yung et al., 1999)
Arrestin	Arr2	(Vicente-Sanchez et al., 2018; Cheng et al., 1998)	(Cheng et al., 1998)	(Manabe et al., 2019)	(Mittal et al., 2013)
	Arr3	(Molinari et al., 2010)	(Morgenweck et al., 2015)	(Molinari et al., 2010; Mori et al., 2017)	(Malfacini et al., 2015)

REFERENCES

Burford, N. T., Tolbert, L. M., and Sadee, W. (1998). Specific G protein activation and μ-opioid receptor internalization caused by morphine, DAMGO and endomorphin I. *European Journal of Pharmacology* 342, 123–126. doi:10.1016/S0014-2999(97)01556-2

Chan, J. S. C., Chiu, T. T., and Wong, Y. H. (2002). Activation of Type II Adenylyl Cyclase by the Cloned μ-Opioid Receptor: Coupling to Multiple G Proteins. *Journal of Neurochemistry* 65, 2682–2689. doi:10.1046/j.1471-4159.1995.65062682.x

Cheng, Z.-J., Yu, Q.-M., Wu, Y.-L., Ma, L., and Pei, G. (1998). Selective Interference of β-Arrestin 1 with κ and δ but Not μ Opioid Receptor/G Protein Coupling. *Journal of Biological Chemistry* 273, 24328–24333. doi:10.1074/jbc.273.38.24328

Hampson, R. E., Mu, J., and Deadwyler, S. A. (2000). Cannabinoid and Kappa Opioid Receptors Reduce Potassium K Current via Activation of G_s Proteins in Cultured Hippocampal Neurons. *Journal of Neurophysiology* 84, 2356–2364. doi:10.1152/jn.2000.84.5.2356

Inoue, A., Raimondi, F., Kadji, F. M. N., Singh, G., Kishi, T., Uwamizu, A., et al. (2019). Illuminating G-Protein-Coupling Selectivity of GPCRs. *Cell* 177, 1933–1947.e25. doi:10.1016/j.cell.2019.04.044

Lou, L.-G., Ma, L., and Pei, G. (1997). Nociceptin/Orphanin FQ Activates Protein Kinase C, and This Effect Is Mediated through Phospholipase C/Ca2+Pathway. *Biochemical and Biophysical Research Communications* 240, 304–308. doi:10.1006/bbrc.1997.7654

Malfacini, D., Ambrosio, C., Gro', M. C., Sbraccia, M., Trapella, C., Guerrini, R., et al. (2015). Pharmacological Profile of Nociceptin/Orphanin FQ Receptors Interacting with G-Proteins and β-Arrestins 2. *PLOS ONE* 10, e0132865. doi:10.1371/journal.pone.0132865

Manabe, S., Miyano, K., Fujii, Y., Ohshima, K., Yoshida, Y., Nonaka, M., et al. (2019). Possible biased analgesic of hydromorphone through the G protein-over β-arrestin-mediated pathway: cAMP, CellKey™, and receptor internalization analyses. *Journal of Pharmacological Sciences* 140, 171–177. doi:10.1016/j.jphs.2019.06.005

Mittal, N., Roberts, K., Pal, K., Bentolila, L., Fultz, E., Minasyan, A., et al. (2013). Select G-Protein-Coupled Receptors Modulate Agonist-Induced Signaling via a ROCK, LIMK, and β-Arrestin 1 Pathway. *Cell Reports* 5, 1010–1021. doi:10.1016/j.celrep.2013.10.015

Molinari, P., Vezzi, V., Sbraccia, M., Grò, C., Riitano, D., Ambrosio, C., et al. (2010). Morphine-like Opiates Selectively Antagonize Receptor-Arrestin Interactions. *Journal of Biological Chemistry* 285, 12522–12535. doi:10.1074/jbc.M109.059410

Morgenweck, J., Frankowski, K. J., Prisinzano, T. E., Aubé, J., and Bohn, L. M. (2015). Investigation of the role of βarrestin2 in kappa opioid receptor modulation in a mouse model of pruritus. *Neuropharmacology* 99, 600–609. doi:10.1016/j.neuropharm.2015.08.027

Mori, T., Kuzumaki, N., Arima, T., Narita, M., Tateishi, R., Kondo, T., et al. (2017). Usefulness for the combination of G protein- and β-arrestin-biased ligands of μ-opioid receptors: Prevention of antinociceptive tolerance. *Molecular Pain* 13, 174480691774003. doi:10.1177/1744806917740030

Narita, M., Ohsawa, M., Mizoguchi, H., Aoki, T., Suzuki, T., and Tseng, L. (2000). Role of the phosphatidylinositol-specific phospholipase C pathway in -opioid receptor-mediated antinociception in the mouse spinal cord. *Neuroscience* 99, 327–331. doi:10.1016/S0306-4522(00)00202-5

Offermanns, S. and Simon, M. I. (1995). $G\alpha_{15}$ and $G\alpha_{16}$ Couple a Wide Variety of Receptors to Phospholipase C. *Journal of Biological Chemistry* 270, 15175–15180. doi:10.1074/jbc.270.25.15175

Preto, A. J., Barreto, C. A. V., Baptista, S. J., Almeida, J. G. d., Lemos, A., Melo, A., et al. (2020). Understanding the Binding Specificity of G-Protein Coupled Receptors toward G-Proteins and Arrestins: Application to the Dopamine Receptor Family. *Journal of Chemical Information and Modeling* 60,

3969–3984. doi:10.1021/acs.jcim.0c00371

Sandhu, M., Touma, A. M., Dysthe, M., Sadler, F., Sivaramakrishnan, S., and Vaidehi, N. (2019). Conformational plasticity of the intracellular cavity of GPCR-G-protein complexes leads to G-protein promiscuity and selectivity. *Proceedings of the National Academy of Sciences* , 201820944doi:10.1073/pnas.1820944116

Szücs, M., Boda, K., and Gintzler, A. R. (2004). Dual Effects of DAMGO [d-Ala^2,*N*-Me-Phe^4,Gly^5-ol]-Enkephalin and CTAP (D-Phe-Cys-Tyr-D-Trp-Arg-Thr-Pen-Thr-NH_2) on Adenylyl Cyclase Activity: Implications for μ-Opioid Receptor G_s Coupling. *Journal of Pharmacology and Experimental Therapeutics* 310, 256–262. doi:10.1124/jpet.104.066837

Sánchez-Blázquez, P., Gómez-Serranillos, P., and Garzón, J. (2001). Agonists determine the pattern of G-protein activation in μ-opioid receptor-mediated supraspinal analgesia. *Brain Research Bulletin* 54, 229–235. doi:10.1016/S0361-9230(00)00448-2

Vicente-Sanchez, A., Dripps, I. J., Tipton, A. F., Akbari, H., Akbari, A., Jutkiewicz, E. M., et al. (2018). Tolerance to high-internalizing δ opioid receptor agonist is critically mediated by arrestin 2. *British Journal of Pharmacology* 175, 3050–3059. doi:10.1111/bph.14353

Wang, D., Zeng, J., Li, Q., Huang, J., Couture, R., and Hong, Y. (2016). Contribution of adrenomedullin to the switch of G protein-coupled μ-opioid receptors from Gi to Gs in the spinal dorsal horn following chronic morphine exposure in rats: Adrenomedullin induces uncoupling of receptor to Gi protein. *British Journal of Pharmacology* 173, 1196–1207. doi:10.1111/bph.13419

Yung, L. Y., Joshi, S. A., Chan, R. Y., Chan, J. S., Pei, G., and Wong, Y. H. (1999). GalphaL1 (Galpha14) couples the opioid receptor-like1 receptor to stimulation of phospholipase C. *The Journal of Pharmacology and Experimental Therapeutics* 288, 232–238

Zhu, T., Fang, L.-y., and Xie, X. (2008). Development of a universal high-throughput calcium assay for G-protein-coupled receptors with promiscuous G-protein G15/16. *Acta Pharmacologica Sinica* 29, 507–516. doi:10.1111/j.1745-7254.2008.00775.x

3 SUPPLEMENTARY FIGURES

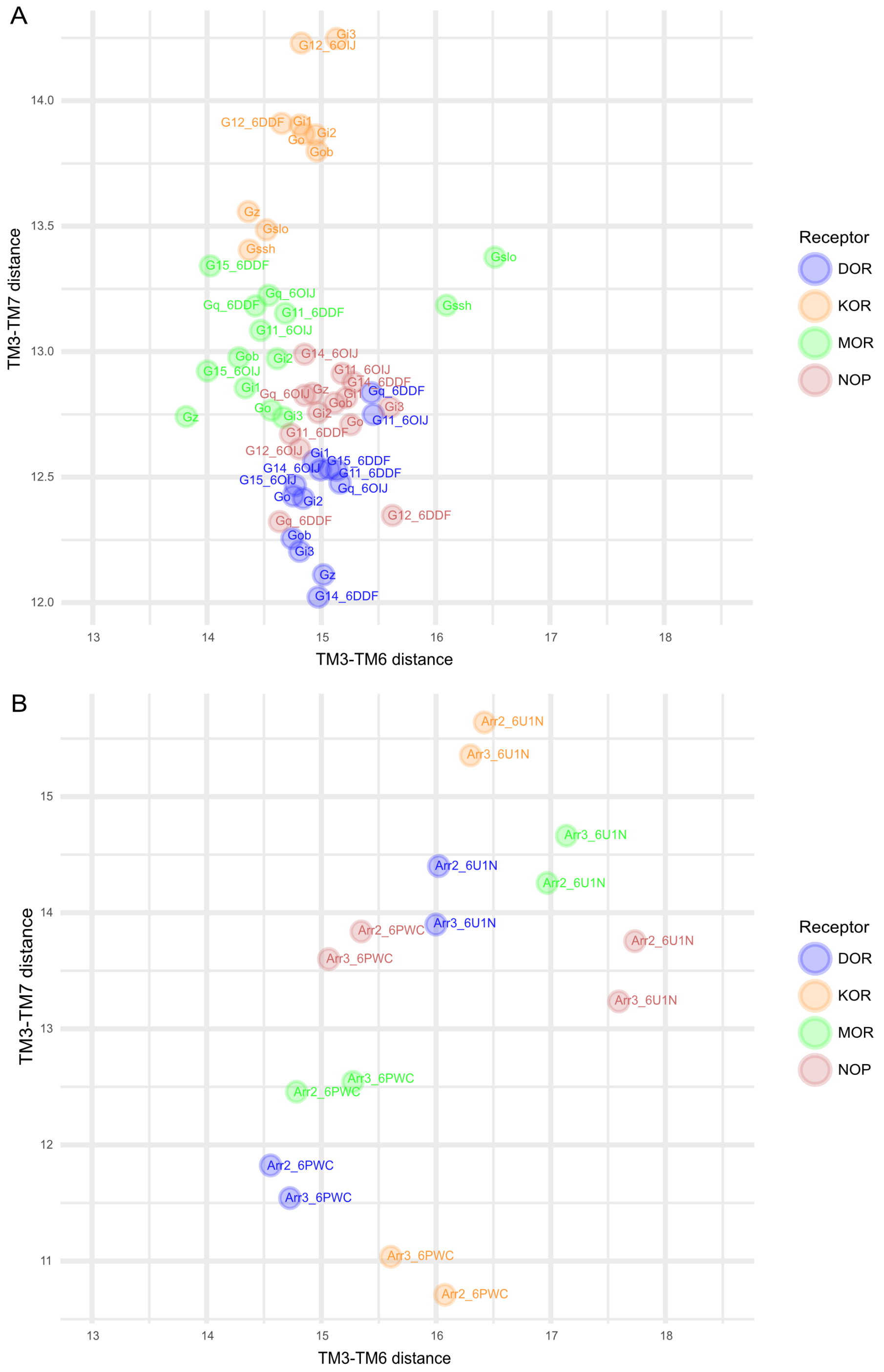

Figure S1. Interhelical distances: A - G-protein and B - Arrestin-coupled models.

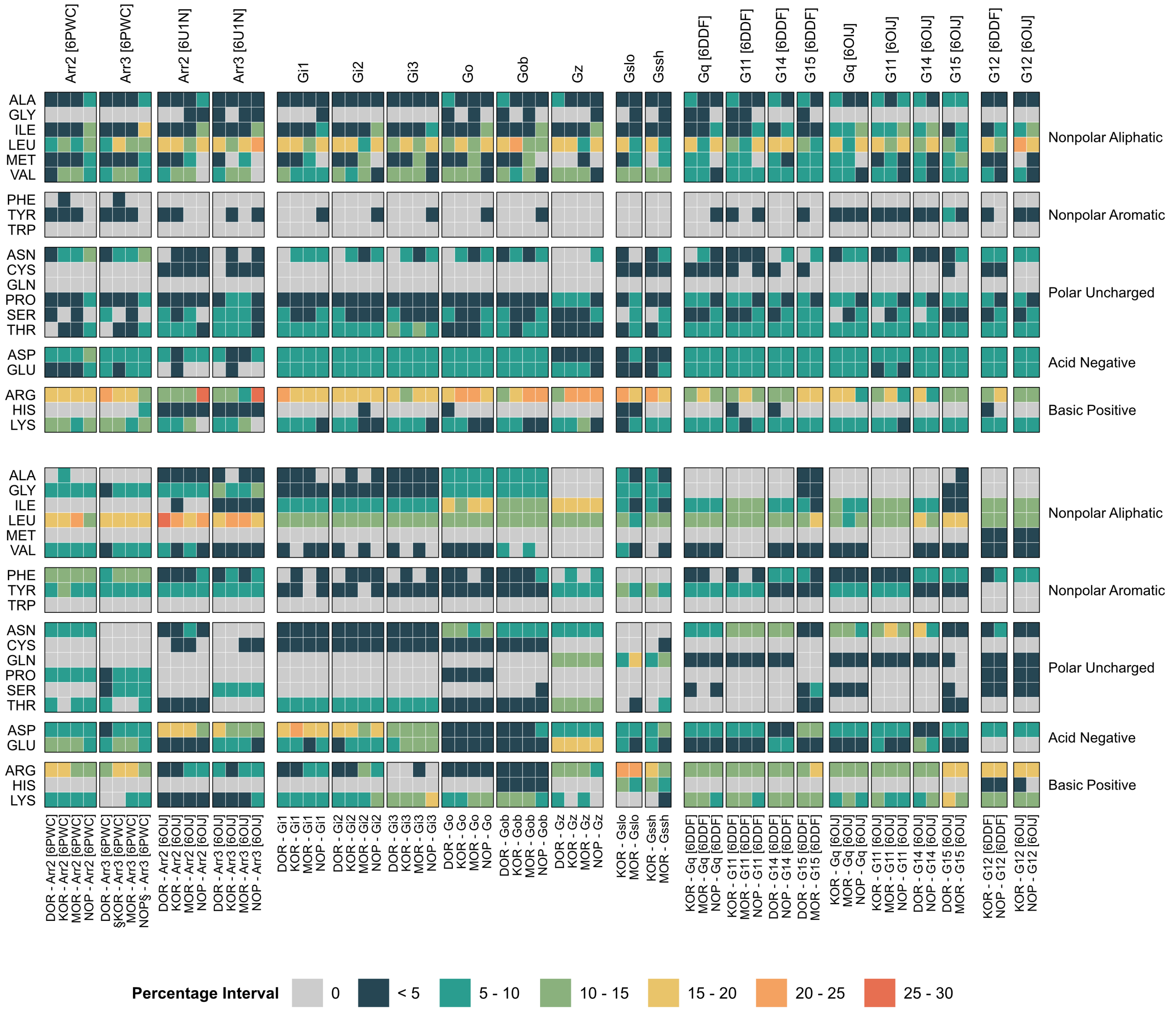

Figure S2. Interfacial amino acid percentage, gouped by partner: Top panel - in receptor side and Bottom panel - partner side.

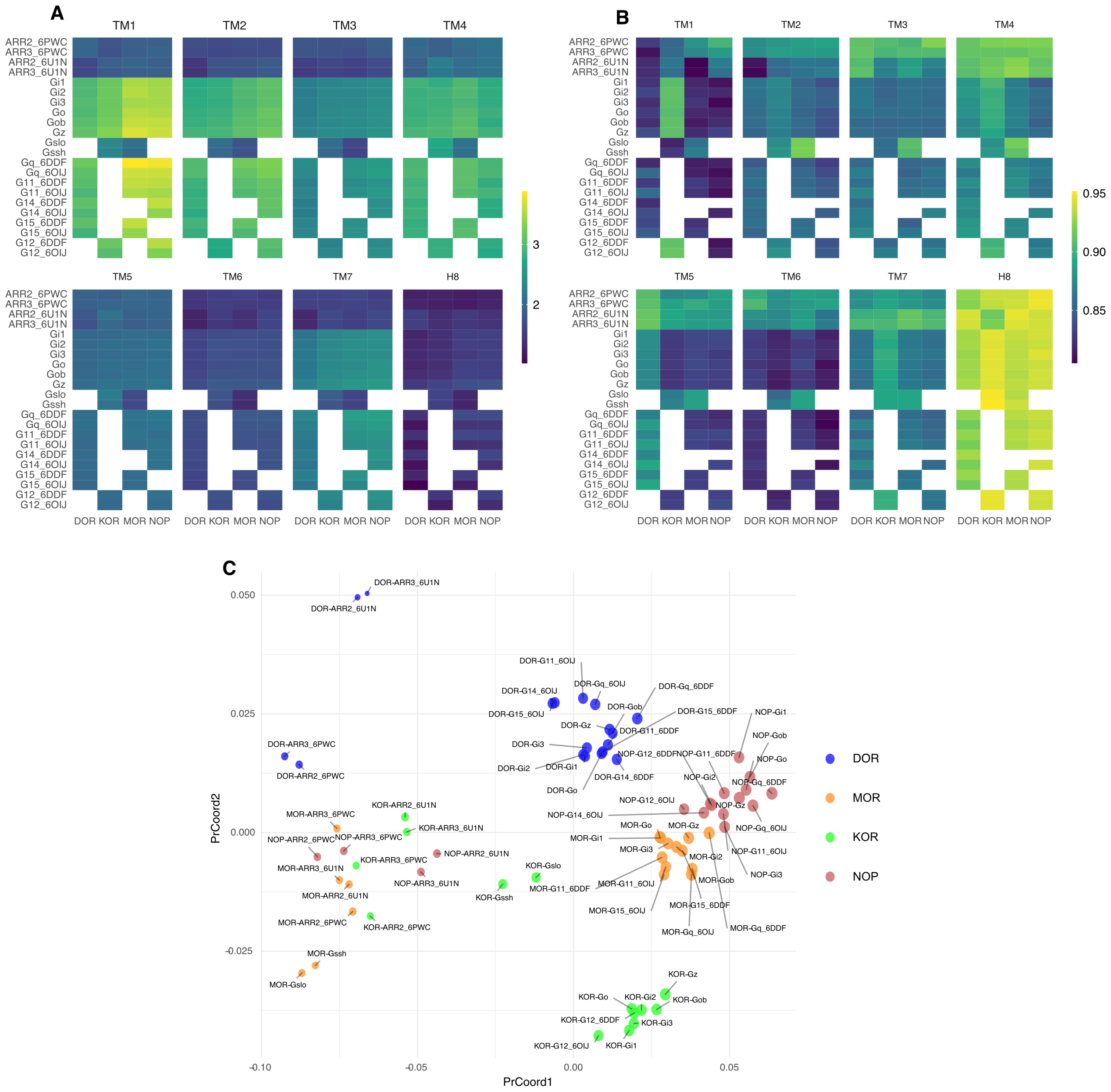

Figure S3. Dynamical analysis of OR complexes. (A) Average fluctuation fold changes for all OR substructures, between OR in monomer and in complex forms. (B) Flexibility changes for all OR substructures, measured through Bhattacharyya distance between OR in monomer and in complex forms. (C) Multidimensional scaling of the flexibility change values. The dots size are directly proportional to average fluctuation fold change values.
